# Beak and skull shapes of human commensal and non-commensal house sparrows *Passer domesticus*

**DOI:** 10.1186/1471-2148-13-200

**Published:** 2013-09-17

**Authors:** Sepand Riyahi, Øyvind Hammer, Tayebeh Arbabi, Antonio Sánchez, Cees S Roselaar, Mansour Aliabadian, Glenn-Peter Sætre

**Affiliations:** 1 Department of Biology, Faculty of Sciences, Ferdowsi University of Mashhad, Mashhad, Iran; 2 Current address: Evolutionary Ecology Associate Research Unit (CSIC), Natural History Museum of Barcelona, Passeig Picasso s/n, Barcelona 08003, Spain; 3 Natural History Museum, University of Oslo, Blindern, P. O. Box 1172, Oslo N-0318, Norway; 4 Institute of Pharmacy and Molecular Biotechnology, Department of Biology, Im Neuenheimer Feld 364, Heidelberg D-69120, Germany; 5 Catalan Institute of Paleontology, Campus de la UAB, Cerdanyola del Vallès 08193, Spain; 6 Naturalis Biodiversity Center, Vertebrate Department, Darwinweg 2/PO Box 9517, RA Leiden 2300, Netherlands; 7 Centre for Ecological and Evolutionary Synthesis (CEES), Department of Biological Sciences, University of Oslo, Blindern, P. O. Box 1066, Oslo N-0316, Norway

**Keywords:** Geometric morphometrics, Beak shape, Granivorous bird, Passer domesticus, Human commensalism

## Abstract

**Background:**

The granivorous house sparrow *Passer domesticus* is thought to have developed its commensal relationship with humans with the rise of agriculture in the Middle East some 10,000 years ago, and to have expanded with the spread of agriculture in Eurasia during the last few thousand years. One subspecies, *P. d. bactrianus,* residing in Central Asia, has apparently maintained the ancestral ecology, however. This subspecies is not associated with human settlements; it is migratory and lives in natural grass- and wetland habitats feeding on wild grass seeds. It is well documented that the agricultural revolution was associated with an increase in grain size and changes in seed structure in cultivated cereals, the preferred food source of commensal house sparrow. Accordingly, we hypothesize that correlated changes may have occurred in beak and skull morphology as adaptive responses to the change in diet. Here, we test this hypothesis by comparing the skull shapes of 101 house sparrows from Iran, belonging to five different subspecies, including the non-commensal *P. d. bactrianus*, using geometric morphometrics.

**Results:**

The various commensal house sparrow subspecies share subtle but consistent skeletal features that differ significantly from those of the non-commensal *P. d. bactrianus*. Although there is a marked overall size allometry in the data set, the shape difference between the ecologically differentiated sparrows cannot be explained by differences in size alone. Relative to the size allometry commensal house sparrows exhibit a skull shape consistent with accelerated development (heterochrony), resulting in a more robust facial cranium and a larger, more pointed beak.

**Conclusion:**

The difference in skull shape and robustness of the beak between commensal and non-commensal house sparrows is consistent with adaptations to process the larger and rachis encapsulated seeds of domesticated cereals among human associated populations.

## Background

The avian beak and associated traits have long been popular targets for studies of adaptive trait evolution. The beak is the main food-processing tool of a bird. Hence, its size and shape are expected to be chiselled by natural selection to fit the feeding ecology of its carrier. One celebrated example of adaptive beak evolution is the radiation and associated diversification of feeding niches and beak shapes among Darwin’s finches [1,2]. Long-term field studies of these finches have provided strong evidence that beak morphology evolves rapidly in response to changing ecological conditions, such as food type, food availability and interspecific competition [2-4]. Morphological evolution may be constrained by various factors, however [5]. For instance, the size and shape of morphological traits in animals may be strongly constrained by body size and may exhibit more or less fixed allometric size relationships across species [6,7]. Yet, the avian beak appears to be a trait that often exhibits high levels of evolvability [8-11].

The house sparrow *Passer domesticus* is a granivorous, widely distributed, human associated bird species [12,13]. The origin of its commensal relationship with humans has been debated for some time [14-16]. However, in a recent paper Sætre *et al*. [17] used population genetic data, as well as previously published fossil and ecological data, to suggest that the house sparrow became associated with early human agricultural societies that rose in the Middle East about 10,000 years ago and then experienced a massive size and range expansion from about 4,000 years ago as agricultural civilizations spread through the Palearctic and Oriental regions. Although the house sparrow comprises several phenotypically distinct subspecies, these are virtually identical at neutral genetic markers, suggesting that phenotypic differentiation is of very recent origin [17].

One of the subspecies has apparently maintained the ancestral ecology of the species, however. The subspecies *P. d. bactrianus* is not associated with humans but breeds in natural or seminatural habitats, such as riverine scrub, hedgerows and trees near pastures and grassland, often far from human habitation [17,18]. It is similar to other house sparrows at neutral genetic markers, however, consistent with the hypothesis that the ecological difference is associated with a recent ecological transition of the commensal house sparrows [17]. In autumn *P. d. bactrianus* move southwards in large flocks from their breeding grounds in Central Asia to reach their wintering grounds in south-east Iran and western parts of the Indian sub-continent [19-21]. Significant for this study, *P. d. bactrianus* feed mainly on wild grass seeds whereas the commensal subspecies prefer seeds from cultivated crops, such as wheat and oats [12]. The evolution of bony structures is possibly less constrained by weight in sedentary than in migratory birds because they are less dependent on endured flight. Hence, the abandonment of migratory behaviour in commensal house sparrows may have facilitated any evolutionary trend towards increased size and robustness of the beak and skull structures.

The abandonment of migratory behaviour in commensal house sparrows is likely to be an adaptation to the year-round supply of food provided by sedentary human societies through storage of cereals, spilling and feeding of domestic animals [17]. Further, the transition from a diet of mainly wild seeds to one consisting mainly of grains from cultivated cereals [12] is likely to have imposed selection pressures on beak and skull morphology of the sparrows. Domestication of plants is associated with an increase in seed size [22-25], but domesticated cereals also differ from their wild relatives in several other traits, such as seed hardness and texture [26]. Moreover, domesticated cereal seeds remain encapsulated in a tough rachis that holds the seed together, whereas in wild cereals the rachis fragmentizes at ripening [24]. These trait differences would most certainly affect seed processing in a granivorous bird and hence the optimal size and shape of its beak and skull. In Darwin’s finches handling of larger and harder seeds is associated with larger, deeper beaks and a correlated increase in robustness of the muscle and jaw architecture of the birds [27].

Here, we apply geometric morphometrics to study variation in the beak and skull of commensal and non-commensal house sparrows from the same overall geographic region, namely Iran. The diversity of house sparrow subspecies is high in Iran (five recognized subspecies, including the non-commensal *P. d. bactrianus*) [12]. Moreover, climatic and environmental conditions vary extensively regionally, from the moist and fertile temperate shore of the Caspian Sea in the north, through dry steppes and deserts in central parts, to tropical habitats in the south of the country. Climatic conditions are likely to affect agricultural practice, the choice of which crop species to cultivate and seed characteristics, all of which may affect optimal beak and skull morphology [11]. However, cultivated crops differ from their wild ancestors in consistent ways [24], suggesting that adaptations to commensalism per se may have larger effects on morphological evolution than such local climatic variation. If adaptations to cultivated cereals have played a significant role in the recent morphological evolution of the house sparrow, we expect to find relatively larger divergence between commensal and non-commensal sparrows in beak and skull morphology than among the various commensal house sparrow subspecies, despite extensive environmental variation. We further predict that the commensal types should share (derived) skeletal features. Finally, we specifically predict that commensal house sparrows would have larger, more robust beaks and skull features associated with a more robust muscle and jaw architecture compared to the non-commensal *P. d. bactrianus*, as adaptations to process the larger and tougher seeds of cultivated cereal crops. At the proximate level, accelerated development (heterochrony) is a well-characterized ontological process that one could predict would yield the necessary changes in robustness.

## Methods

**Figure 1:**
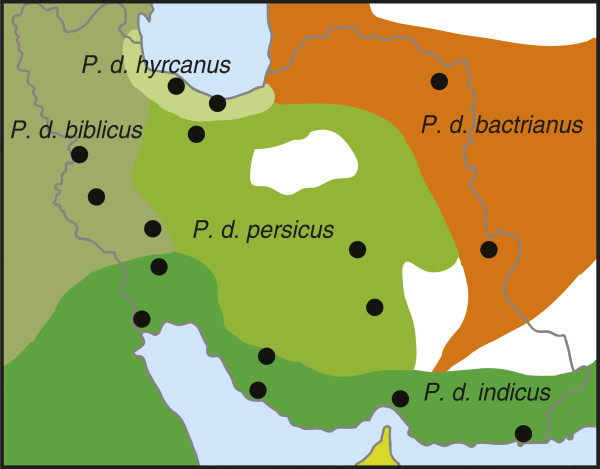
**Geographic distribution of four commensal subspecies of the house sparrow (green tones) and the non-commensal *P. d. bactrianus *(orange) in Iran.** Sampling locations are indicated with black dots.

101 completely ossified adult skulls from the house sparrow were collected from 16 populations in Iran (Figure 1). The specimens represent five of the eleven house sparrow subspecies described by Summers-Smith [12] that have been defined based on differences in plumage coloration, size and ecology; the non-commensal and migratory *P. d. bactrianus* (*N* = 12) and the four commensal subspecies *P. d. biblicus* (*N* = 33), *P. d. persicus* (*N* = 30), *P. d. hyrcanus* (*N* = 12) and *P. d. indicus* (*N* = 14). The specimens were obtained from different museum and university collections in Iran.

For geometric morphometrics on bird skulls and beaks, landmark analysis has been the most common approach (e.g. [28]). For this study, we instead applied outline analysis of the skull shapes. Outlines may be better adapted to complex shapes having important shape variation contained in smooth areas between any well-defined homologous landmarks. For cranial shapes, outline analysis can outperform landmark analysis in terms of statistical discrimination power [29]. Outline analysis circumvents the subjective selection of shape features inherent to landmark analysis. Finally, outline analysis allows visualization of statistical results as full biological shapes rather than as deformation grids or truss networks of a small collection of landmarks.

Digital images (dorsal view) were prepared using an Olympus DP71i camera connected to an Olympus BX51 stereomicroscope (Olympus Corporation, Tokyo, Japan). The camera was fixed to a custom made rack that ensured that the skulls where photographed from the same distance and 90˚ angle. The skulls were fixed on a plasticine platform and care was taken to orient the skull consistently relative to the camera. Any residual random distortion is expected to produce unbiased noise. To quantify morphological variation the x- and y-coordinates of each skull outline were digitized using the tpsDig 2.2 software [30]. The starting point of the outline was defined as the tip of the bill. 150 roughly equally spaced points were measured on each outline (the subsequent shape analysis does not require equal spacing).

**Figure 2:**
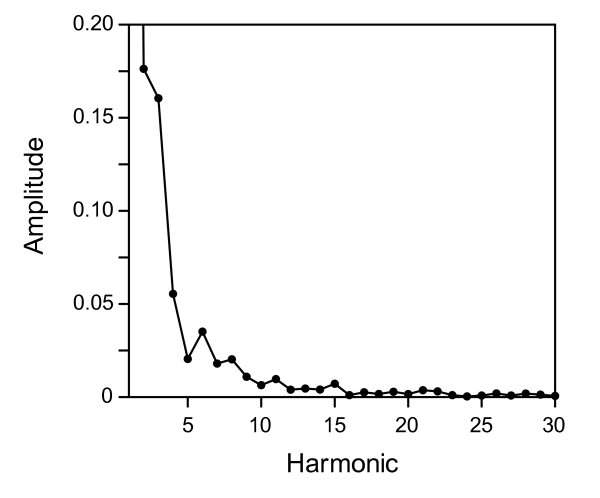
**The average Elliptic Fourier Analysis spectrum across all specimens.** The amplitude of each harmonic is calculated as the Euclidean norm of its four coefficients. The amplitude of the first harmonic (not shown) is 1.11.

Elliptic Fourier Analysis, EFA [31] is a well-established technique for outline analysis. The procedure involves spectral (Fourier) decomposition of the *x* and *y* increments along the contour. Each harmonic is represented by four coefficients, i.e. the cosine and sine amplitudes of Δ*x* and Δ*y*. This is equivalent to decomposition into a sum of harmonically related ellipses. EFA was carried out in the software Past, version 2.07 [32], with the “invariant to starting position and rotation” option. Size was recorded by the length of the semimajor axis of the first harmonic ellipse [31]. After this calculation, size was removed prior to further EFA analysis, to ensure that the EFA coefficients themselves contained only shape variation, in order to reduce high-frequency noise and limit the number of variables. A plot of the average EFA spectrum (Figure 2) showed very small amplitudes beyond 15 harmonics and we thus chose this as the cut-off point for inclusion in the analyses.

The resulting 60 coefficients were subjected to further multivariate analysis in Past. For testing of multivariate group means we used a permutation test, PerMANOVA, [33] based on Euclidean distance. For regression of shape on size we used ordinary least squares multivariate regression with MANOVA testing. For tests on allometry in commensal versus non-commensal subspecies we included size, commensality and size-commensality interaction as independent variables.

The allometric trajectories of the different subspecies were also investigated using the Common Allometric Component (CAC) method of Mitteroecker et al. [34]. The CAC is a vector in shape space computed by regression of shape variables (with centered species means) on log size. Additional residual shape components, orthogonal to the CAC, are calculated by PCA on the residuals after CAC regression. The resulting shape vectors were visualized by generating synthetic skull outlines in Past, version 3.0.

## Results

### Size and shape allometry

**Figure 3:**
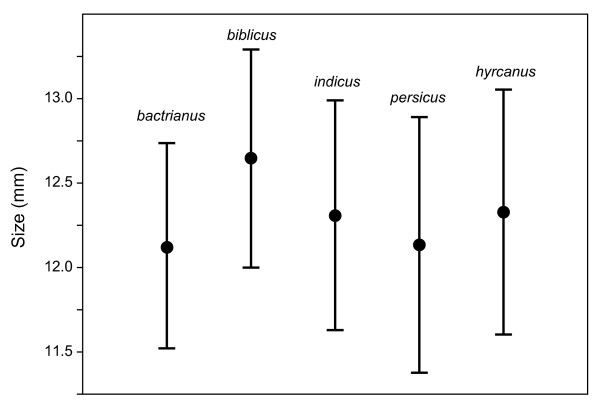
**Sizes of skulls from the five subspecies of *P. domesticus *(means and two standard deviations).***P. d. bactrianus* is the only non-commensal subspecies.

The sizes (Figure 3) differed only slightly between the non-commensal *P. d. bactrianus* with mean size 12.13 mm and the four commensal subspecies with pooled mean size 12.38 mm (*t* = 2.00, *p* = 0.048). However, there was considerable size variation among the subspecies (one-way ANOVA, *F* = 9.85, *p* < 0.001) and particularly *P. d. biblicus* stood out as larger than *P. d. bactrianus* (Tukey’s *Q* = 5.97, *p* = 0.00063) and *P. d. persicus* (*Q* = 5.91, *p* = 0.00072).

**Figure 4:**
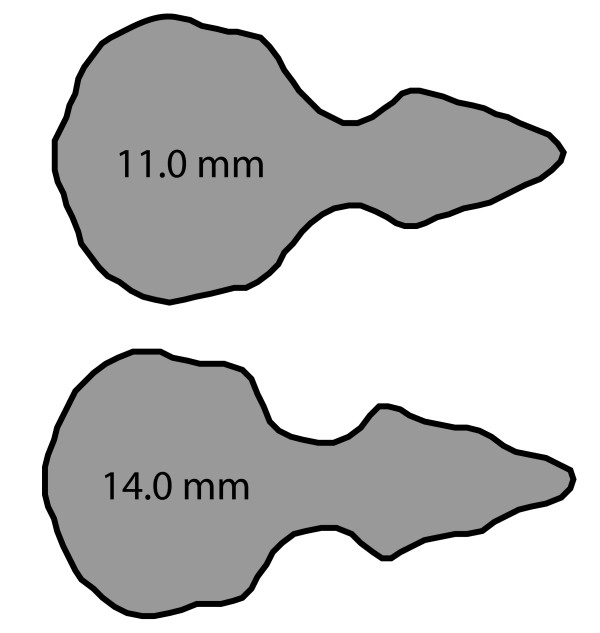
**Synthetic (predicted) skull shapes for two different skull sizes, based on linear regression of the first 15 EFA harmonics upon size.** The skulls are normalized to equal size.

A multivariate regression of EFA coefficients with size as independent variable was highly significant (Wilks’ *λ* = 0.22, *F* = 2.39, *p* = 0.0021), demonstrating clear size allometry for the data pooled over all subspecies. No obvious nonlinearity or heteroskedasticity was observed in the regression, and the data were therefore left untransformed. Figure 4 shows the shape predicted from the regression for semimajor axis sizes of 11 and 14 mm. The cranial shape predicted for a size of 11 mm (S11) show some morphological differences with regard to the one predicted for a size of 14 mm (S14): (a) S14 have relatively smaller braincases than S11, (b) the interorbital width should be more reduced proportionately in S11 sparrows than in S14 ones, and (c) the analysis also predicts a more significantly developed facial cranium (splachnocranium), and a longer bill in particular, in S14 compared to S11 individuals.

### Shape differences between subspecies

**Figure 5:**
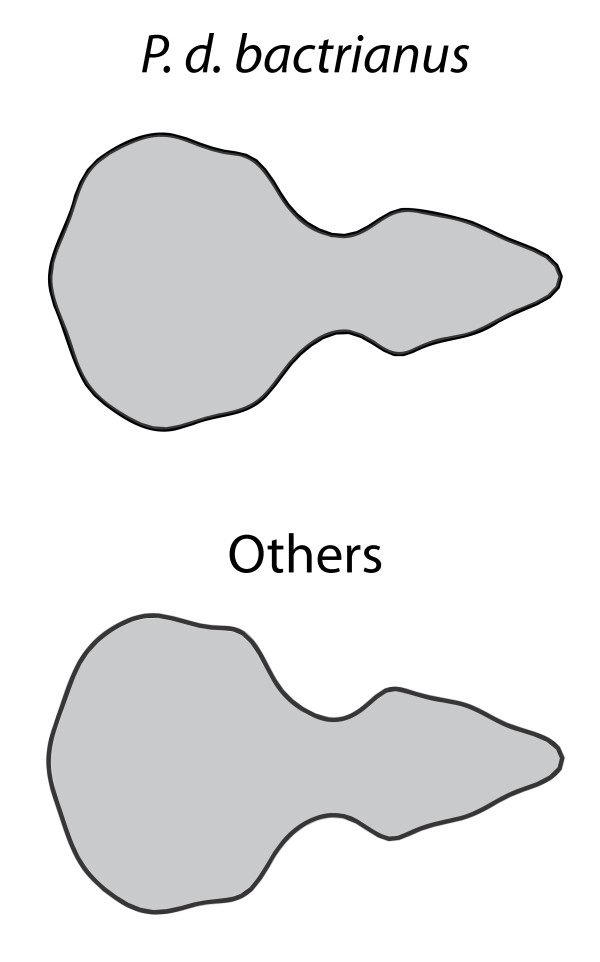
**Average shape of the non-commensal *P. d. bactrianus *versus the commensal subspecies.** Sizes are normalized.

The EFA coefficients of the five subspecies are significantly different (one-way PerMANOVA, *F* = 1.89, *p* = 0.023). To visualize the shape difference between *P. d. bactrianus* and the four commensal subspecies, we show the average (consensus) shapes from the two groups in Figure 5. The commensal subspecies are associated with a more robust facial skeleton and a larger beak. The axis of shape variation in Figure 5 is visually similar to the size allometric axis shown in Figure 4. Accordingly, there is positive correlation between the vector of differences between group means and the size regression slopes (*R* = 0.32, *p* < 0.001).

Multivariate, multiple regression of shape with size, commensality and interaction as independent variables was highly significant (Wilks’ *λ* = 0.020, *F* = 1.85, *p* < 0.001). The analysis showed that shape was significantly dependent on all of size (*λ* = 0.26, *F* = 1.94, *p* = 0.014), commensality (*λ* = 0.29, *F* = 1.68, *p* = 0.043) and size-commensality interaction (*λ* = 0.29, *F* = 1.65, *p* = 0.048). In other words, there is both an overall size allometry and a difference in allometric trajectories between commensal and non-commensal subspecies.

**Figure 6:**
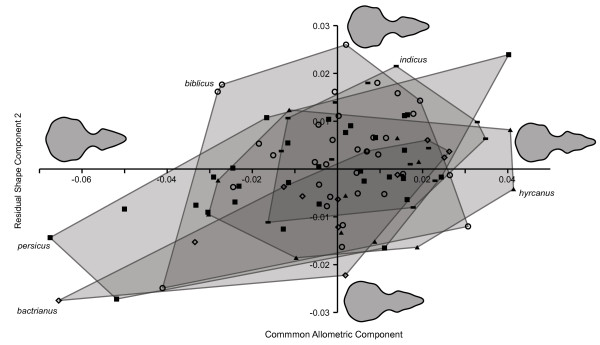
**CAC versus RSC 2 scores for the five subspecies of *P. domesticus*.** Reconstructed skull outlines are shown in their respective positions in CAC-RSC 2 space.

A plot of the scores on the Common Allometric Component axis and the second Residual Shape Component (RSC 2, explaining 18% of residual shape variation) is shown in Figure 6. The first RSC (RSC 1, 33%) was found to describe left-right asymmetry, and showed no structure with respect to taxonomy. The visualization of overall size allometry (CAC) confirms the result shown in Figure 4, obtained similarly by regression but with linear size and without within-group centering. RSC 2 describes shape variation mainly restricted to the orbital area, with higher scores associated with narrower interorbital area and more pronounced postorbital processes. Although there is considerable overlap between the subspecies, the non-commensal *P. d. bactrianus* occupies a region of particularly low scores on RSC 2. It may also be noted that *P. d. bactrianus*, and to some extent the similarly small-sized *P. d. persicus*, shows correlation between CAC and RSC 2, not apparent in the other subspecies.

**Figure 7:**
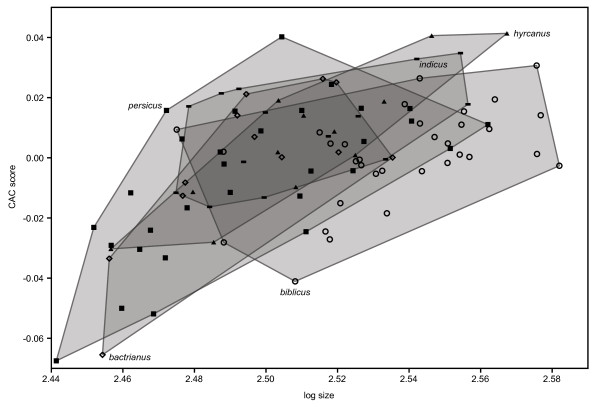
**Log semimajor axis size versus CAC scores for the five subspecies of *P. domesticus*.**

Further details are apparent when plotting size against CAC (Figure 7). The different subspecies show some vertical displacement along the CAC axis, which could be referred to as heterochrony in a very loose sense, although the differences in allometric trajectories make this term less appropriate [34]. In addition, the larger subspecies *P. d. indicus* and *P. d. biblicus* have smaller slopes in the size-CAC plot than the smaller three subspecies.

**Figure 8:**
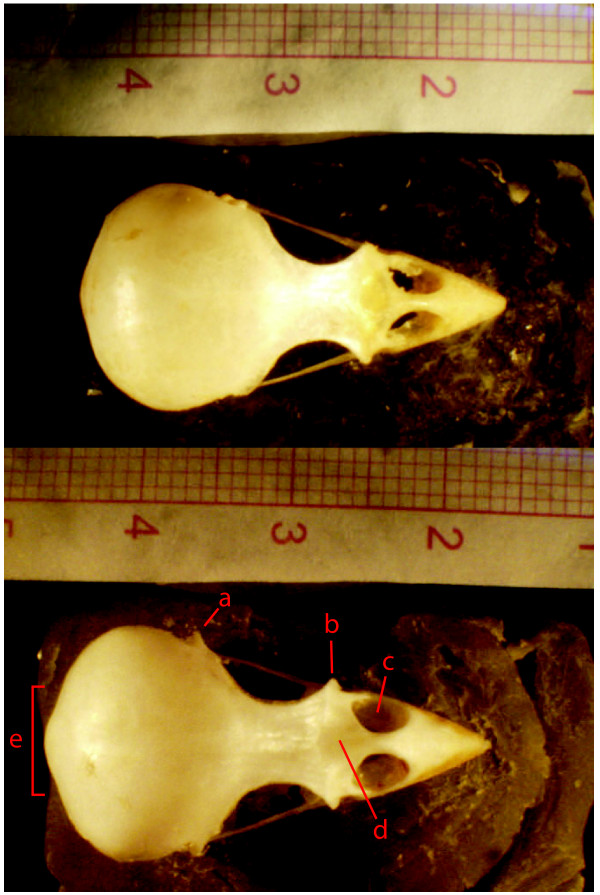
**Photograph (dorsal view) of a non-commensal house sparrow *P. d bactrianus *(upper panel) and a similar sized commensal house sparrow *P. d. persicus *(lower panel).** Notice the difference in robustness and elongation of the facial cranium, and in particular **a)** the postorbital process, **b)** the lachrymal process, **c)** the oral surface of the maxillare as seen through the nostrils, **d)** the nasal process and **e)** the occipital region.

The morphological differences described statistically above and depicted in Figure 5 are easily visible when inspecting the actual bone structures of the different skulls. In Figure 8 we show the skull of a non-commensal *P. d. bactrianus* and a commensal house sparrow (*P. d. persicus*). *P. d. persicus* was chosen because it is the subspecies most similar in size to *P. d. bactrianus*. The chosen pictures were otherwise randomly selected. The skull of *P. d. persicus* appears overall more robustly constructed than the skull of *P. d. bactrianus*, and the splachnocranium and the beak are both elongated and more heavily built. The postorbital process (a) and the lachrymal (b) are more expanded laterally in *P. d. persicus* than in *P. d. bactrianus* (cf. Figure 6). Although not included in the statistical analysis above since the features do not affect the outline of the skull, it also appears that the oral surface of the maxillare, as one can see through the nasal openings, is wider (c), and that the nasal process of the rostrum (d) is larger in *P. d. persicus* compared to *P. d. bactrianus.* Differences in the occipital region (e) are also consistent with increased robustness in the commensal subspecies (Figure 6).

## Discussion

We demonstrate subtle but consistent morphological differences in the skull and beak of commensal and non-commensal house sparrows. Across subspecies residing in different environments commensal house sparrows have consistently more robust facial craniums and larger, more pointed beaks compared to the non-commensal *P. d. bactrianus*. We further found a clear, overall allometric relationship between skull size and shape, larger skulls having a relatively smaller brain case and a larger, more robust facial cranium than smaller ones. The commensal house sparrows were on average slightly larger than the non-commensal *P. d. bactrianus*, but the difference in cranial shape persisted after controlling statistically for size. The morphological changes thus appear consistent with accelerated development (heterochrony) of commensal house sparrows, assuming that the more “juvenile-like” skull of *P. d. bactrianus* represents the ancestral state as suggested by a previous study [17]. Our statistical analyses indicate a significant interaction between size and ecology (commensal vs. non-commensal). An allometric shift like the one demonstrated here is often considered a hallmark of an adaptive morphological change (e.g. [35,36]). Additionally however, we found an orthogonal shape variation of commensality only, suggesting a relatively complex ontology in the development of beak and skull shape in the case of these sparrows. When plotting size against Common Allometric Component (Figure 7) there appears to be a gradient from the smaller subspecies to the larger ones with decreasing slopes but increasing intercepts. This may be referred to as a heterochronic pre-displacement, but note that strictly speaking, heterochrony is defined with respect to age and not size.

The house sparrow is primarily a seed eater and vegetable matter makes up 85-90% of the food, complimented with more protein rich food of animal origin, particularly in the diet provided to the nestlings [37]. Although commensal house sparrows can be quite opportunistic in what they eat they are regarded as specialized on cultivated cereal crops and will prefer oats and wheat when there is a choice [12]. The larger and rachis encapsulated seeds of domesticated cereals [24,26] are therefore likely to have changed the optimal feeding apparatus of the commensal house sparrows in the direction of increased robustness.

Many of the skeletal features of the commensal house sparrows as compared to the non-commensal *P. d. bactrianus* can be understood as adaptations to process the larger and tougher seeds of domesticated cereals. The lachrymal and postorbital processes (both larger in commensal sparrows) are connected to the posterior region of the mandible through ligaments [38]. The attachment of these ligaments together with the musculus depressor mandibulae, play an important role in the protraction of the upper jaw [39]. The musculus depressor mandibulae, which depresses the lower jaw, originates in the occipital region of the cranium (again more developed in commensal sparrows) and inserts in the mandible [40]. Another important muscle, the aductor mandibulae externus has its origin in a wide area of the posterior part of the skull, passes forwards and downwards between the postorbital process, and inserts in the posterior part of the mandible [41]. The chewing area of the beak is stronger (more bony-built) in commensal house sparrows than in *P. d. bactrianus* and the larger nasal process in the commensal sparrows would provide more robustness in the kinetic area between the beak and the rest of the skull.

## Conclusions

In conclusion, our results are consistent with the hypothesis that adaptations to food sources provided by human societies, and particularly cultivated cereals, have shaped the beak and skull of commensal house sparrows to become more robust and the facial cranium and beak to become elongated and more pointy. In a future study it would be interesting to study the feeding ecologies of the non-commensal *P. d. bactrianus* and the various commensal subspecies in greater detail in order to link ecology and morphology more directly. Further, it would be interesting to compare the anatomy of fossil house sparrow skulls with the various contemporary ones. House sparrow fossils are relatively common in archaeological excavation sites [42] as are cereal seeds [24]. Hence, in principle, it should be possible to trace evolutionary changes in the morphology of the beak and skull of the house sparrow through time, from the rise of agriculture and beyond. Combining such studies with ontological observations may further improve our understanding of the developmental changes that has taken place as the house sparrow beak and skull became adapted to process cultivated cereal grains.

## Competing interests

The authors declare that they have no competing interests.

## Authors’ contributions

MA conceived of the study, data was collected by SR and TA under supervision of MA, analysis and interpretation of results were done by SR, ØH and GPS, the manuscript was written by GPS, ØH and SR with contributions from AS and CSR. All authors read and approved the final manuscript.
